# Coefficients of variation of ground reaction force measurement in cats

**DOI:** 10.1371/journal.pone.0171946

**Published:** 2017-03-29

**Authors:** Eva Schnabl-Feichter, Alexander Tichy, Barbara Bockstahler

**Affiliations:** 1 Department for Companion Animals and Horses, University Clinic for Small Animals, Small Animal Surgery, University of Veterinary Medicine, Vienna, Austria; 2 Department for Biomedical Science, Platform Bioinformatics and Biostatistics, University of Veterinary Medicine, Vienna, Austria; 3 Department for Companion Animals and Horses, University Clinic for Small Animals, Small Animal Surgery, Section for Physical Therapy, University of Veterinary Medicine, Vienna, Austria; Colorado State University, UNITED STATES

## Abstract

Gait analysis has been extensively performed in dogs and horses; however, very little is known about feline biomechanics. It was, therefore, the aim of this study to determine the coefficient of variation (CV) among three ground reaction force (GRF) measurements taken for 15 client-owned European shorthaired cats without a training period and a short acclimatisation time. Gait was measured as each cat walked across a pressure-sensitive walkway, and measurements were made three times over a multi-week period (range: 2 to 17 weeks). The parameters evaluated were peak vertical force (PFz), vertical impulse (IFz), stance phase duration (SPD), step length (SL), paw contact area (PCA) and symmetry index (SI%) of the front and hind limbs. After averaging each of the values from the three measurements, the CV and 95% confidence interval (CI) were calculated for all parameters. PFz showed the lowest CV (~ 3%), while IFz showed the highest CV (~11%) when normalised to body mass. When the GRFs were normalised to total force, the CV of PFz dropped to ~2% and that of IFz dropped to ~3%. The CV of SL and PCA were lower (~6% respectively ~5%) compared to the CV for SPD (~10%). The SI% for both PFz and IFz were comparable to the values reported in the gait analysis literature for dogs. Results of the current study indicate that gait analysis of cats using pressure-sensitive walkways produces reliable data and is a promising approach for evaluation of lameness. The results also suggest that PFz may be a more reliable parameter than IFz and that normalisation to percent of total force may aid in interpretation of the evaluated data.

## Introduction

Biomechanical motion analysis has been a fundamental aspect of orthopaedic research for many decades. Various motion analysis techniques are available to describe normal and disturbed locomotion and to investigate the impact of treatment modalities. Among the most well established motion analysis techniques are ground reaction force (GRF) measurement, joint and spine kinematics, and electromyography (EMG); however, the technique most frequently used to describe normal and disturbed locomotion is kinetic gait analysis.

The gold standard to measure GRFs are single or multiple force plates embedded in stationary walkways [1, 2] or treadmills [3, 4]. An alternative to this motion analysis technique are pressure-sensitive walkways (PSWs) [5, 6]. The PSW usually have a high number of pressure sensors to facilitate quantification of both high and low pressure areas, vertical forces, and temporal characteristics of the stance phase upon coming into contact with the paw [7, 8].

The parameters evaluated in kinetic gait analysis using force plates include orthogonal GRFs that result from contact between the paw and the ground during gait, such as the mediolateral force and craniocaudal force, and the vertical forces of peak vertical force (PFz) and vertical impulse (IFz). Additional parameters that are evaluated include rate of loading and temporal gait characteristics. Paw pressure distributions can be only measured with PSWs [9]. Among all of these parameters, the PFz and IFz are the most commonly evaluated [10].

Kinetic gait analysis has been widely performed in canines and equines, and is well established in these species. However, very little information on feline kinetics and kinematic biomechanics has been reported in the publicly available literature. Among the few reports on felines, the data have shown that, like other quadrupeds, cats exert greater forces in their forelimbs, making them a hind limb-driven species. The investigations using PSWs have shown PFz for the forelimbs as ranging between 48.2% and 62.0% Bodymass (BM) [11–15] and for the hind limbs as ranging between 38.3% and 50.2% BM [11, 13, 15], and IFz for the fore limbs as ranging between 12.7% and 18.9% BM [11–15] and for the hind limbs as ranging between 13.1% and 14.6% BM [11, 13, 15]. Moreover, in the study of distribution of vertical force within feline paws, Stadig and Bergh [16] showed that the mean weight during a strike is transferred from the caudal towards the craniomedial part of the paw.

It is well recognized that cats are inherently more difficult to work with in studies of gait analysis than dogs; indeed, most of the previously performed studies report that acclimatisation and training was a prerequisite to gaining objective data [13, 15]. Moreover, the reliability of replicable measures of GRF in cats on PSW remains unknown. Therefore, we designed this study of GRFs in client-owned cats using PSWs to include repeated measurements and using only a very short acclimatisation time with no training period, in order to determine whether reproducible results are achievable, as evidenced by calculations of the coefficient of variation (CV) among the three GRF measurements over various multi-week periods.

## Materials and methods

### Animals

This study was carried out with pre-approval given by the institutional ethics committee of the Veterinary University Vienna/Austria in accordance with Good Scientific Practice guidelines and national legislation (reference number 21/01/97/2014). Client-owned European shorthair cats were recruited to the study, for voluntary participation. A board certified surgeon performed orthopaedic and clinical examinations of all recruited cats. X-rays of the hips, stifles and elbows were done following standard procedures protocols. Any sign of visible lameness or pain elicited during the orthopaedic examination, of abnormality detected during the clinical exam, or of osteoarthritis in any of the joints radiographed precluded study participation. Of the 15 cats that met the criteria for study inclusion, 7 were neutered males and 8 were spayed females, with a mean body mass (± SD) of 5.5 ± 1.2 kg (range: 4–6.6 kg) and mean age of 7.2 ± 4.2 years (range: 2.6–14.9 years).

### Experimental setting and technical equipment

The study was performed as a non-randomized prospective trial. Sample size calculation was based on previous published results of vertical force measurements. Using known baseline values for sound cats, we estimated the hind limb PFz to be 50.8 ± 6.8% BM, and using those for cats with coxarthrosis we estimated the hind limb PFz to be 42.8 ± 5.6% BM [17]; accordingly, a total number of 15 cats was calculated as necessary to detect a difference between the measurements with a one-tailed alpha of 0.05 and a power of 0.80, based on a cut-off value of 5% for the discrimination of PFz values between measurement days.

All examinations and measurements were performed at the University of Veterinary Medicine, Vienna. For the gait analysis, a Zebris FDM Type 2 pressure plate (Zebris Medical GmbH, Allgäu, Germany) was mounted in the middle of a 7 m runway. The pressure plate was 203.2 x 54.2 cm in size and included 7040 sensors with a sampling rate of 100 Hz. The plate was covered with a rubber mat to hide the measuring area from the cats’ sight and prevent slipping. The gait analysis study was conducted in a quiet room, with only the owner and the research examiner present.

Upon arrival for each gait analysis study, each cat was taken out of the transport box and allowed a few minutes of free movement in the examination room to facilitate acclimatisation prior to initiation of the study. Before we started the measurement, the PSW was calibrated with the rubber mat in place according to the manufactures instructions. For the study, the cat was first enticed by toys, food, verbal and visual stimuli to cross the pressure-sensitive mat in a straight line. In addition, a movable cartoon wall was placed beside the plate depending on the positive response of each cat. Each cat was allowed to determine their own walking pace over the pressure plate, and the measurement consisted of at least five valid steps cycles (i.e. walking in a straight line with the head in a straightforward position, without an apparent change of velocity).

Measurements were repeated three times within 2–17 weeks. While cats were allowed to walk at their own comfortable pace, only trials with a difference in the gait velocity of ≤ 0.3 m/s within the same individual were accepted for further analysis. Each measurement trial was video recorded using a Panasonic NV-MX500 camera, and data were stored using WinFDM software (v1.2.2; Zebris Medical GmbH) and processed using the specially developed software (Pressure Analyzer 1.3.0.2; Michael Schwanda).

### Data processing and outcome parameters

Parameters under evaluation were PFz, IFz, step length (SL; m), paw contact area (PCA; cm^2^), stance phase duration (SPD; s) and SI (symmetry index, %) of the front and hind limbs.

The SI of the contralateral limb pair of each measurement day was calculated using the formula:

$$SIXFz=abs\left(\frac{\left(XFzFL-XFzFR\right)}{\left(XFzFL+\;XFzFR\right)}\right)\times 100$$

where: SI = symmetry index, X = the mean value of PFz or IFz from one measurement day for each cat, FL = front left, FR = front right, hind limb symmetry was calculated accordingly; a SI of 0% would represent perfect symmetry between the contralateral limb pair.

To calculate the overall symmetry index the mean of the SI of each measurement day and each cat was calculated.

Gait velocity was recorded from the left forelimb. The GRF data, initially displayed in Newtons (N), were first normalized to the cats’ body mass (expressed as % BM) and then normalized to the sum of all forces exerted by the four limbs (expressed as % total force [TF]).

### Statistical methods

All data were processed with IBM SPSS statistical software, version 19. The Kolmogorov-Smirnov test was used to determine normal distribution. Descriptive statistics were calculated for each parameter at each of the three measurement days and averaged. Data are given as mean and SD in the paper and for better comparison of GRFs with other already valuable data as mean and SD and also SEM in the tables or as indicated in the text. The lower and upper 95% confidence interval (CI) was also reported for all parameters. An ANOVA with Bonferroni post-hoc test was used to detect differences between the repeated GRF measurements. Pearson’s correlation was used to assess the relation between mean GRF (N, in % BM and in % TF) and BM, SL, SPD and PCA. The coefficient of variation (CV) over the three average measurements was calculated as the SD relative to the mean of the measurement for each parameter. A *p*-value < 0.05 was considered statistically significant.

## Results

**Fig 1:**
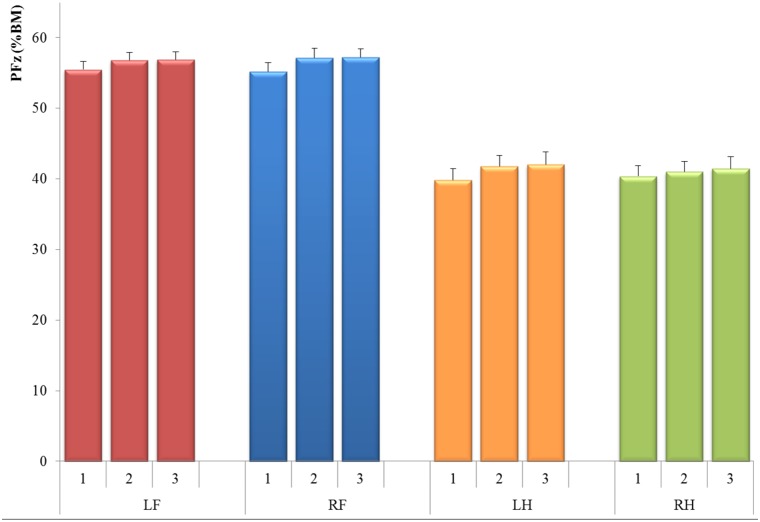
Mean values for three measures of peak vertical force (PFz) for all 15 tested cats and all tested legs, normalized to % Body Mass (BM). Limbs: LF = Left fore, RF = Right fore, LH = Left hind, RH = Right hind.

**Fig 2:**
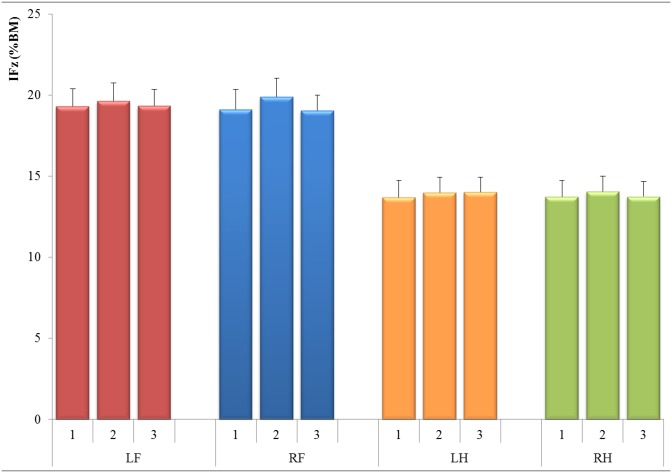
Mean values for three measures of vertical impulse (IFz) for all 15 tested cats and all tested legs, normalized to % Body Mass (BM). Limbs: LF = Left fore, RF = Right fore, LH = Left hind, RH = Right hind.

The gait velocity measured for the cats ranged between 0.39 m/s and 0.83 m/s (mean ± SD: 0.64 ± 0.09 m/s). The mean PFz- and IFz-values (for all four legs with corresponding CIs) over the three measurement days are shown in Table 1, where PFz %BM was around 56% for the front, and 40% for the hind limbs. IFz %BM was around 19% for the front, and 14% for the hind limbs. The PFz and IFz (mean ± SEM) showed great homogeneity among the 15 tested cats and among the three measurement days; as illustrated in Figs 1 and 2.

**Table 1:** Mean values for three measures of gait analysis of all 15 tested cats and all tested legs.

Limb, laterality	Mean	± SD	± SEM	UCI 95%	LCI 95%	CV, %	± SD	± SEM	Limb, laterality	Mean	± SD	± SEM	UCI 95%	LCI 95%	CV, %	± SD	± SEM
**Fore, left**									**Fore, right**								
PFz, % BM	56.41	3.95	1.02	58.41	54.41	3.77	1.89	0.49	PFz, % BM	56.53	4.37	1.13	58.74	54.32	3.92	1.69	0.44
PFz, % TF	28.98	2.25	0.58	30.12	27.84	1.36	0.85	0.22	PFz, % TF	29.03	2.29	0.59	30.19	27.87	1.51	1.07	0.28
IFz, % BM	19.42	3.69	0.95	21.29	17.56	11.56	6.53	1.69	IFz, % BM	19.35	3.80	0.98	21.28	17.43	13.18	5.14	1.33
IFz, % TF	29.37	2.24	0.58	30.50	28.23	2.77	1.38	0.36	IFz, % TF	29.23	2.30	0.59	30.39	28.06	2.99	1.43	0.37
SPD, s	0.48	0.08	0.02	0.52	0.44	9.41	6.16	1.59	SPD, s	0.48	0.08	0.02	0.52	0.44	9.99	5.41	1.40
SL, m	0.49	0.05	0.01	0.51	0.46	5.46	3.44	0.89	SL, m	0.48	0.04	0.01	0.51	0.46	5.10	2.96	0.76
PCA, cm^2^	12.57	1.42	0.37	13.29	11.85	4.50	2.81	0.72	PCA, cm^2^	12.66	1.32	0.34	13.32	11.99	4.76	3.31	0.85
**Hind, left**									**Hind, right**								
PFz, % BM	41.21	6.22	1.61	44.36	38.06	3.82	2.77	0.72	PFz, % BM	40.97	5.65	1.46	43.83	38.11	3.77	3.03	0.78
PFz, % TF	21.06	2.49	0.64	22.32	19.80	2.45	1.13	0.29	PFz, % TF	20.93	2.10	0.54	22.00	19.87	2.80	1.79	0.46
IFz, % BM	13.90	3.39	0.87	15.62	12.19	13.27	6.02	1.56	IFz, % BM	13.84	3.39	0.88	15.56	12.13	13.12	6.64	1.72
IFz, % TF	20.74	2.22	0.57	21.87	19.62	3.39	1.84	0.48	IFz, %TF	20.66	2.33	0.60	21.84	19.48	4.21	1.90	0.49
SPD, s	0.46	0.07	0.02	0.49	0.42	10.34	5.14	1.33	SPD, s	0.45	0.07	0.02	0.49	0.42	9.97	5.58	1.44
SL, m	0.50	0.05	0.01	0.53	0.47	6.08	3.86	1.00	SL, m	0.50	0.05	0.01	0.52	0.47	6.20	3.95	1.02
PCA, cm^2^	12.24	2.22	0.57	13.36	11.12	3.95	2.30	0.59	PCA, cm^2^	12.20	2.09	0.54	13.26	11.15	5.63	2.60	0.67

Mean = Mean of the three measurements, SD = Standard deviation, SEM = Standard error of the mean, CV = Coefficient of variation, UCI 95% = Upper border of the 95% confidence interval, LCI 95% = Lower border of the 95% confidence interval, BM = Body mass, TF = Total force, PFz = Peak vertical force, IFz = Vertical impulse, SPD = Stance phase duration, SL = Step length, PCA = Paw contact area.

### Coefficient of variation

**Fig 3:**
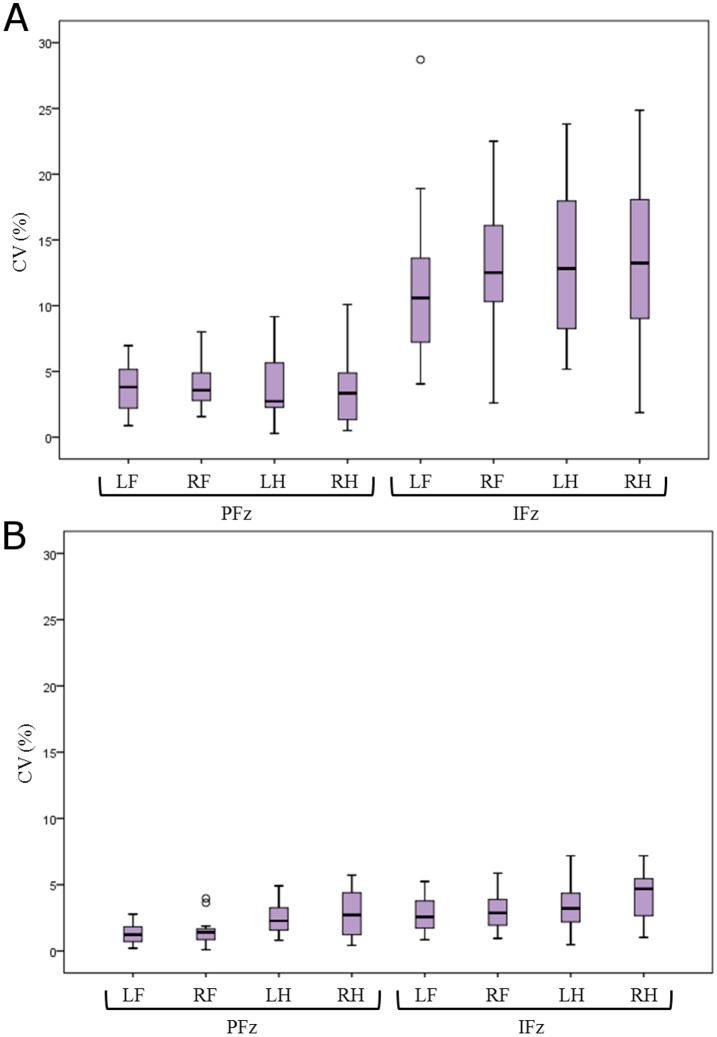
Coefficient of variation for the three measures with (A) normalisation to Bodymass (%BM) and (B) normalisation to Total Force (% TF). Limbs: LF = Left fore, RF = Right fore, LH = Left hind, RH = Right hind; Parameters: PFz = Peak vertical force, IFz = Vertical impulse; Measures: CV = Coefficient of variation.

PFz was displayed as very reliable data, with a mean CV in a range of 3.77 to 3.92% when normalized to the BM (Fig 3a) and a range of 1.36 to 2.80% when normalized to TF (Fig 3b). In contrast, the IFz was much more instable, with a mean CV ranging between 11.56% and 13.27% when normalized BM (Fig 3a) and 2.77% to 4.21% when normalized to TF (Fig 3b). The coefficient of variation of SPD was in a range of 9.41 to 10.34%, of SL in a range of 5.1–6.2% and the CV of PCA ranged from 3.95 to 5.63% (Tbl 1).

### Differences between measurement days

There were no significant differences found to exist between any of the three measurements for SI, PFz, IFz, BM, SL, or SPD. The only exception was PCA, where the GRF left hind limb was significantly higher at measurement day 2 compared to day 1 (12.39 ± 0.57 cm^2^ vs. 12.0 ± 0.62 cm^2^, *p* = 0.013).

### Correlations

**Fig 4:**
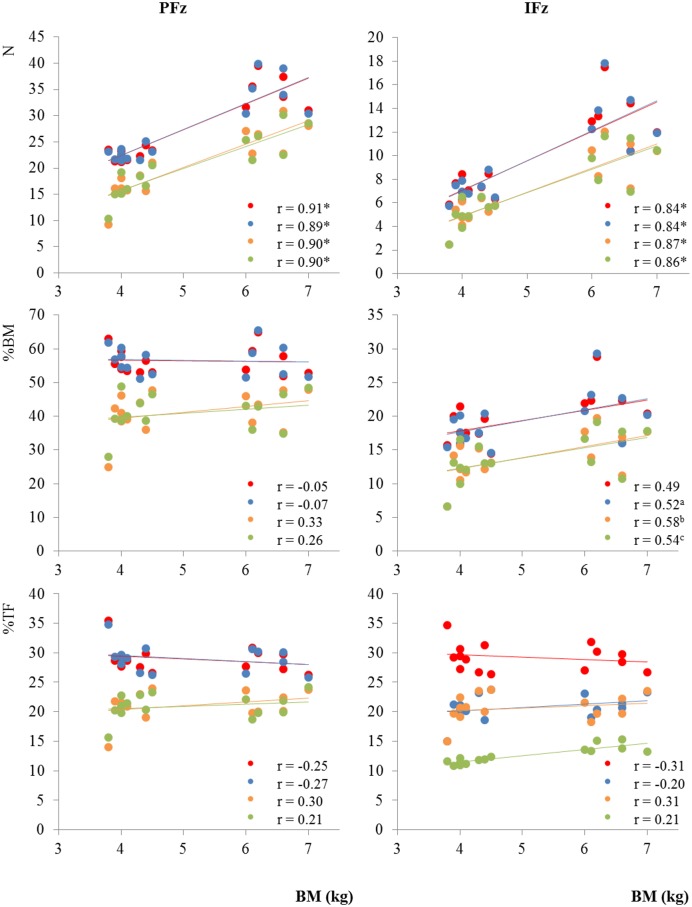
Ground reaction force measurements correlated to the body mass. Limbs: Red: Left fore, Blue: Right fore, Orange: Left hind, Green: Right hind; Parameters: PFz = Peak vertical force, IFz = Vertical impulse; Measures: BM = Body mass, TF = Total force; * = p = 0.00; ^a^ = 0.05, ^b^ = 0.02, ^c^ = 0.04.

BM did not correlate with SL, but significant correlations were found for BM with SPD (*r* for all legs > 0.58, *p* ≤ 0.02) and with PCA (*r* for all legs > 0.87, *p* = 0.00). However, BM was significantly correlated with both un-normalized (in Newtons) PFz (*r* for all legs > 0.89, *p* = 0.00) and IFz (*r* for all legs > 0.84, *p* = 0.00). When the GRF were normalized to % BM, the correlation with BM was lost for PFz (*r* ranging from -0.05 for the left fore limb to 0.33 for the left hind limb, *p* > 0.24) and was decreased for IFz (*r* ranging from 0.49 for the left fore limb to 0.58 for the left hind limb, *p* < 0.05, where the left fore limb just felt out of significance, *p* = 0.06). When the GRF were normalized to % TF, the correlation with BM was lost for PFz (*r* ranging from -0.27 for the right fore limb to 0.30 for the right hind limb, *p* > 0.28) and for IFz (*r* ranging from -0.20 for the right fore limb to 0.31 for the left hind limb, *p* > 0.26) (Fig 4). Finally, SL and SPD did not show significant correlation (*r* for all legs < 0.31, *p* > 0.26), but PCA did show a significant correlation to SPD (*r* for all legs > 0.57, *p* < 0.02) but not to SL (*r* for all legs < 0.44, *p* > 0.1).

## Discussion

This study determined the CV for GRF measurements of domestic cats taken on three days. By taking measurements on separate days and without providing any training period we were able to attain a homogenous dataset from the multi-day study design. We calculated the CV to test the reliability of measurements taken in this multi-day approach. In general, one can say that the larger the CV, the less reliable the data is. In our study, the CVs for PFz normalized to % BM and to % TF, as well for SL and PCA, were small, indicating reliability. The CV for IFz had a broader range, up to 26.61% when normalised to % BM, as did the CV for SPD when compared to the lower value obtained when normalized to % TF. These results suggest that GFR data displaying larger CV, when normalised to % BM, should be interpreted carefully.

Voss et al. [2] reported that standard normalisation of GRF measurements to BM or body weight (BW) was insufficient to account for differences in body size and relative velocity of dogs. The authors therefore postulated normalisation of GRF measurements, especially for the time-associated variables like IFz and SPD, to BW and body size instead. For our study described herein, we considered the findings reported by Strasser et al. [6] and chose to normalise the GRFs PFz and IFz to % BM and to % TF, and showed that the CV of IFz became more reliable when normalized to % TF. We chose to not perform normalisation of the data to body size because we had used a homogenous group of domestic shorthaired cats and did not expect any large differences in body size; future studies using cats with different morphometric parameters, such as the Maine Coon domesticated breed, should be performed in order to more accurately investigate the possible influence of body height on GRF.

GRF measurement is a well-established procedure in gait analysis of horses and dogs. In contrast, it has not been frequently applied to studies of cats. A recent review summarized the data reported in the publicly available literature on measurements of PFz and IFz in cats, which had been obtained mainly through use of pressure-sensitive mats. All of the studies included in the review had allowed for a period of acclimatisation or performed training prior to the testing [18]. But training on a leash, as it has been applied in a recent study [19] is not in general applicable in client owned cats. Our study, however, showed that even without an extensive acclimatisation period or training period reliable results could be produced in a uniform population of domestic shorthaired cats. This achievement was likely reliant on the fact that we only included cats walking in a straight line without instances of pausing or trotting. Moreover, we had also excluded measurements taken when a cat turned their head during the recorded gait cycle, in consideration of the data reported by Stadig and Bergh [16] which showed an increase in PFz of the fore limbs related to the side that the head was positioned towards.

In our study, the recorded speeds varied between 0.39 and 0.83 m/s, which represented a range that is comparable to those reported for other related studies [13, 15, 16]. Lascelles et al. [13], which had evaluated cats at gaits between 0.37 and 0.83 m/s, found no significant difference between kinetic parameters measured at speeds below and above 0.6 m/s. The results for PFz and IFz gained are also comparable to the data from previous studies [15, 20]. One point to consider is that GRF data from force plates and PSW differs, as values derived from PSW seem to be lower than those derived from force plates [7]. It is also important to note here that the difference we observed between the fore and hind limbs, and in other reported PFz and IFz measurements as well, are nevertheless useful to discriminate cats as quadruped animals that are fore limb-dominant and hind limb-driven.

The SIs of PFz and IFz measured in our cat population are comparable to those reported in dogs [21], which suggests that pressure-sensitive mats can aid in discrimination between lame and non-lame cats, similar to the evaluation approach that has been successfully used with dogs. Indeed, a previous study using such an approach has been reported, in which cats with osteoarthrosis were evaluated before and after non-steroidal anti-inflammatory drug treatment [20]. In the future it will be very interesting to use this approach to examine different pathologies and their related lameness in fore and hind limbs, to investigate compensation mechanisms, and to evaluate treatment response.

PFz was the most stable parameter of the GRFs evaluated in our current study, in agreement with results reported previously by Strasser et al. [6] and Bockstahler et al. [22]. PFz represents a single point on the GRF curve that is generated during the stance phase, in contrast to IFz which is a function of time and force and which represents an overview of all forces exerted during the entire stance phase. The nature of these two measurements should be considered to interpret results of studies in which they are used in the evaluation of treatment effects; for example, the SL and SPD in fore and hind limbs of our study displayed similar results but the former yielded a lower CV. When PFz measurements were normalized to % BM, in our study, there was no correlation between BM and PFz, but there still was a correlation between BM and IFz. This difference between PFz and IFz This can be explained by the fact that IFz is a function of time and force and therefore also depends on SPD. When both parameters were normalized to % TF, however, there was no correlation with BM for either. Therefore, in accordance to Voss et al. [2], we recommend that future studies in cats without lameness should normalize GRFs to % TF. If this can also be applied to cats with lameness should be investigated in other studies.

Limitations of this study exist, and are largely due to the inherent nature of our feline study subjects. It is more difficult to guide cats in a straight line, without any instances of pausing or visible changes in gaits over the plate, than it is to do so with dogs. This distinctive nature of cats could be responsible for the differences we observed in measured velocities, which may in turn have been responsible for the variances observed in our GRF measurements. In future studies it possible would be of benefit to additionally measure acceleration, as it could give more information about variances in the data gained. Indeed, some of our evaluated cats would not cross the mat after the first trial, but they were excluded from analysis.

Ultimately, the discipline of gait analysis in cats requires more time and patience to gain data because of such inherent challenges to study performance.

## Conclusions

Through this study, we have been able to provide more data on normal gait in felines, particularly for a homogenous domestic population of European shorthaired cats measured with a pressure sensitive mat. Results for the PFz and IFz (mean ± SEM of three measurements taken from 15 cats on 3 days) showed substantial homogeneity among the cats and among the different measurement days, with values for PFZ around 56% BM for the fore, and 40% BM for the hind limbs and measurements for IFz % BM around 19% for the fore, and 14% for the hind limbs.

Because our data provided evidence of the reliability of most of the values and measured an SI which is comparable to that reported for dogs, gait analysis using PSWs without an extensive acclimatisation or training period might be a promising approach to lameness evaluation in domestic cats.

## Supporting information

S1 File Raw data. V = Velocity, LF = Left fore limb, RF = Right fore limb, LH = Left hind limb, RH = Right hind limb, SL = Step length, PFz = Peak vertical force, Ifz = Vertical impulse, PCA = Paw contact area, SPD = Stance phase duration, _1 = First measurement day, _2 = Second measurement day, _3 = Third measurement day.
(PDF)

## References

[1] Nordquist BI, Fischer J, Kim SY, Stover SM, Garcia-Nolen T, Hayashi K et al Effects of trial repetition in clinically normal Labrador Retrievers. Vet Comp Orthop Traumatol. 2011; 24: 435–44. 10.3415/VCOT-11-01-0015 21938309

[2] Voss KI, Wiestner T, Galeandro L, Hässig M, Montavon PM. Effect of dog breed and body confirmation on vertical ground reaction forces, impulses and stance times. Vet Comp Orthop Traumatol. 2011; 24: 106–12. 10.3415/VCOT-10-06-0098 21243175

[3] Bockstahler BA, Vobornik A, Müller M, Peham C. Compensatory load redistribution in naturally occuring osteoarthritis of the elbow joint and induced weight-bearing lameness of the forelimbs compared with clinically sound dogs. Vet J. 2009; 180: 202–12. 10.1016/j.tvjl.2007.12.025 18406183

[4] Fischer SI, Andreas A, Nolte I, Schilling N. Compensatory load redistribution in walking and trotting dogs with hind limb lameness. Vet J. 2013; 197: 746–52. 10.1016/j.tvjl.2013.04.009 23683534

[5] Oosterlinck MI, Biosmans T, Gasthuys F, Polis I, Van Ryssen B, Dewulf J et al Accuracy of pressure plate kinetic asymmetry indices and their correlation with visual gait assessment scores in lame and nonlame dogs. Am J Vet Res. 2011; 72: 820–5. 10.2460/ajvr.72.6.820 21627529

[6] Strasser T, Peham C, Bockstahler BA. A comparison of ground reaction forces during level and cross-slope walking in Labrador Retrievers. BMC Vet Res. 2014; 10: 1–8. 25262070 10.1186/s12917-014-0241-4 PMC4181697

[7] Lascelles BD, Roe SC, Smith E, Reynolds L, Markham J, Marcellin-Little D et al Evaluation of pressure walkway system for measurement of vertical limb forces in clinically normal dogs. Am J Vet Res. 2006; 67: 277–82. 10.2460/ajvr.67.2.277 16454633

[8] Souza AN, Pinto AC, Marvulle V, Matera JM. Evaluation of vertical forces in the pads of German Shepherd dogs. Vet Comp Orthop Traumatol. 2013; 26: 6–11. 10.3415/VCOT-11-07-0100 23111688

[9] Gillette RL, Angle TC. Recent developments in canine locomotor analysis: a review. Vet J. 2008; 178, 165–76. 10.1016/j.tvjl.2008.01.009 18406641

[10] McLaughlin RM. Kinetic and kinematic gait analysis in dogs. Vet Clin N Am-Small. 2001; 31: 193–01. 10.1016/s0195-5616(01)50045-5 11787262

[11] Romans CW, Conzemius MG, Horstman CL, Gordon WJ, Evans RB. Use of pressure platform gait analysis in cats with and without bilateral onychectomy. Am J Vet Res. 2004; 65: 1276–8. 15478777 10.2460/ajvr.2004.65.1276

[12] Romans CW, Gordon WJ, Robinson DA, Evans R, Conzemius MG. Effect of postoperative analgesic protocol on limb function following onychectomy in cats. J Am Vet Med Assoc. 2005; 227: 89–93. 16013541 10.2460/javma.2005.227.89

[13] Lascelles BD, Findley K, Correa M, Marcellin-Little D, Roe S. Kinetic evaluation of normal walking and jumping in cats, using a pressure-sensitive walkway. Vet Rec. 2007; 160: 512–6. 17435097 10.1136/vr.160.15.512

[14] Robinson DA, Romans CW, Gordon-Evans WJ, Evans RB, Conzemius MG. Evaluation of short-term limb function following unilateral carbon dioxide laser or scalpel onychectomy in cats. J Am Vet Med Assoc. 2007; 230: 353–8. 10.2460/javma.230.3.353 17269865

[15] Verdugo MR, Rahal SC, Agostinho FS, Govoni VM, Mamprim MJ, Monteiro FOB. Kinetic and temporospatial parameters in male and female cats walking over a pressure sensing walkway. BMC Vet Res. 2013; 9: 129 10.1186/1746-6148-9-129 23803220 PMC3701551

[16] Stadig SM, Bergh AK. Gait and jump analysis in healthy cats using a pressure mat system. J Fel Med Surg. 2015; 17: 523–9. 10.1177/1098612X14551588 PMC10816797 25239912

[17] Guillot M, Moreau M, D'Anjou MA, Martel-Pelletier J, Pelletier JP, Troncy E. Evaluation of Osteoarthritis in Cats: Novel Information from a Pilot Study. Vet Surg. 2012; 41: 328–35. 10.1111/j.1532-950X.2012.00976.x 22380935

[18] Schnabl E, Bockstahler B. Systematic review of ground reaction force measurements in cats. Vet J. 2015; 206: 83–90. 10.1016/j.tvjl.2015.05.017 26118478

[19] Corbee RJ, Maas H, Doornenbal A, Hazewinkel HAW. Forelimb and hindlimb ground reaction forces of walking cats: Assessment and comparison with walking dogs. Vet J. 2014; 202: 116–27. 10.1016/j.tvjl.2014.07.001 25155217

[20] Guillot M, Moreau M, Heit M, Martel-Pelletier J, Pelletier JP, Troncy E. Characterization of osteoarthritis in cats and meloxicam efficacy using objective chronic pain evaluation tools. Vet J. 2013; 196: 360–7. 10.1016/j.tvjl.2013.01.009 23416029

[21] Budsbergh SC, Jevens DJ, Brown J, Foutz TL, DeCamp CE, Reece L. Evaluation of limb symmetry indices, using ground reaction forces in healthy dogs. Am J Vet Res. 1993; 54: 1569–74. 8250378

[22] Bockstahler BA, Tichy A, Aigner P. Compensatory load redistribution in Labrador retrievers when carrying different weights- a non-randomized prospective trial. BMC Vet Res. 2016; 12: 92 10.1186/s12917-016-0715-7 27268096 PMC4897959

