# Supplementary material for: Coefficients of variation of ground reaction force measurement in cats
Source: PLoS One. 2017 Mar 29;12(3):e0171946. doi: 10.1371/journal.pone.0171946 (PMC5371282; doi:10.1371/journal.pone.0171946)
Supplement: S1 File — V = Velocity, LF = Left fore limb, RF = Right fore limb, LH = Left hind limb, RH = Right hind limb, SL = Step length, PFz = Peak vertical force, Ifz = Vertical impulse, PCA = Paw contact area, SPD = Stance phase duration, _1 = First measurement day, _2 = Second measurement day, _3 = Third measurement day. (PDF) [file pone.0171946.s001.pdf]

Bodymass	V_1	V_2	V_3	LF_SL_1	RF_SL_1	LH_SL_1
6	0,39	0,63	0,71	0,39	0,39	0,38
4,1	0,67	0,71	0,69	0,49	0,49	0,48
4,3	0,61	0,53	0,64	0,58	0,50	0,59
3,8	0,80	0,87	0,80	0,51	0,50	0,61
6,1	0,77	0,46	0,60	0,51	0,51	0,55
4	0,63	0,54	0,63	0,44	0,44	0,43
6,6	0,72	0,71	0,61	0,53	0,53	0,57
4,4	0,57	0,73	0,55	0,46	0,47	0,47
4,5	0,81	0,53	0,72	0,46	0,46	0,47
6	0,68	0,62	0,48	0,53	0,55	0,55
3,9	0,58	0,53	0,51	0,46	0,47	0,47
4	0,73	0,79	0,83	0,48	0,50	0,50
6,2	0,53	0,55	0,66	0,51	0,51	0,52
6,6	0,61	0,71	0,77	0,46	0,46	0,46
4	0,51	0,52	0,52	0,44	0,43	0,43

RH_SL_1	LF_SL_2	RF_SL_2	LH_SL_2	RH_SL_2	LF_SL_3	RF_SL_3
0,39	0,46	0,47	0,47	0,47	0,49	0,48
0,48	0,50	0,51	0,52	0,50	0,48	0,52
0,61	0,48	0,49	0,49	0,49	0,51	0,50
0,60	0,54	0,53	0,59	0,59	0,51	0,50
0,53	0,43	0,43	0,45	0,44	0,48	0,49
0,43	0,41	0,40	0,42	0,40	0,44	0,45
0,57	0,53	0,52	0,59	0,57	0,53	0,52
0,46	0,51	0,48	0,42	0,49	0,45	0,46
0,47	0,38	0,40	0,41	0,41	0,44	0,43
0,59	0,62	0,51	0,56	0,62	0,61	0,64
0,47	0,47	0,46	0,46	0,46	0,49	0,49
0,49	0,50	0,48	0,50	0,48	0,54	0,54
0,52	0,52	0,53	0,53	0,54	0,56	0,56
0,46	0,47	0,48	0,59	0,47	0,47	0,48
0,41	0,40	0,45	0,46	0,41	0,41	0,41

**LH_SL_3**	**RH_SL_3**	LF_PFz_1	LF_PFz_2	LF_PFz_3	RF_PFz_1	RF_PFz_2
0,48	0,50	30,58	30,82	31,80	29,86	30,57
0,52	0,47	20,36	21,52	22,60	20,54	21,52
0,50	0,51	22,84	21,97	22,19	21,81	21,29
0,61	0,60	22,69	25,15	22,69	22,23	24,78
0,50	0,51	34,87	35,60	36,18	33,99	35,96
0,44	0,46	21,33	21,79	20,48	21,51	22,51
0,55	0,55	38,32	37,93	36,10	38,33	40,93
0,46	0,46	24,10	24,89	24,26	23,69	26,63
0,45	0,44	23,17	23,34	23,61	22,66	23,12
0,52	0,49	29,54	32,85	32,49	28,30	31,66
0,44	0,52	19,73	20,98	23,04	20,25	21,46
0,55	0,56	23,58	22,37	21,79	24,10	21,31
0,56	0,56	40,17	38,74	39,64	40,25	39,75
0,49	0,49	33,67	34,24	32,89	33,43	34,26
0,43	0,43	21,57	22,98	25,36	22,69	22,78

RF_PFz_3	LH_PFz_1	LH_PFz_2	LH_PFz_3	RH_PFz_1	RH_PFz_2	RH_PFz_3
30,88	26,72	28,40	29,23	27,14	28,59	29,80
23,46	14,53	15,60	17,07	16,06	15,87	16,26
21,63	17,66	19,19	18,97	19,07	17,99	18,47
22,23	8,88	10,02	8,88	10,04	11,09	10,04
35,63	20,88	23,81	23,78	19,77	21,52	23,41
20,18	14,96	15,54	14,80	16,34	16,14	13,59
37,91	30,86	31,71	30,08	29,51	32,28	28,67
25,13	14,80	15,33	16,60	16,20	16,01	17,81
23,61	20,76	21,50	20,90	21,06	19,68	20,75
31,12	26,00	28,25	26,97	23,52	26,53	25,96
23,46	15,26	16,19	17,07	13,90	14,93	16,26
22,71	16,14	15,98	16,14	15,94	15,33	14,20
39,46	26,92	25,60	26,90	27,10	25,42	25,74
34,37	23,19	23,17	21,99	22,46	21,69	23,49
25,61	16,76	17,94	19,73	18,08	18,71	20,72

LF_Ifz_1	LF_Ifz_2	LF_Ifz_3	RF_Ifz_1	RF_Ifz_2	RF_Ifz_3	LH_Ifz_1
14,83	10,87	10,24	13,97	11,10	10,67	12,38
6,89	7,06	7,22	6,47	6,89	6,92	4,45
7,65	7,36	7,05	7,14	7,99	7,07	6,48
5,80	5,98	5,80	5,90	5,43	5,90	2,50
11,48	14,68	13,83	11,05	16,61	13,84	6,46
6,40	7,46	6,81	6,18	8,10	6,51	4,41
14,36	14,47	14,47	15,51	13,52	15,03	11,02
8,50	8,29	8,66	8,25	9,12	9,07	5,04
5,70	7,16	6,24	5,75	7,20	6,37	5,31
10,34	13,54	14,85	9,86	13,46	13,38	8,75
7,03	8,69	7,22	7,17	8,37	6,92	5,01
6,98	5,52	6,07	7,06	5,85	5,94	4,53
18,59	18,24	15,73	19,95	17,93	15,63	13,29
12,15	9,30	9,80	11,71	9,82	9,57	8,54
7,93	7,67	9,70	7,70	6,99	8,98	5,52

LH_Ifz_2	LH_Ifz_3	RH_Ifz_1	RH_Ifz_2	RH_Ifz_3	LF_PCA_1	LF_PCA_2
9,50	9,36	11,93	9,96	9,50	13,09	13,11
4,72	4,96	5,11	4,83	4,60	10,40	11,57
6,57	6,22	6,09	7,07	6,43	11,55	12,02
2,43	2,50	2,56	2,32	2,56	11,30	12,11
9,63	8,76	6,09	8,73	8,96	13,88	12,79
5,02	4,87	4,64	5,50	4,44	11,17	12,20
10,78	11,17	11,19	12,09	11,14	15,48	15,82
4,94	5,80	5,44	5,53	5,97	11,53	12,11
6,71	5,37	5,31	6,36	5,63	12,37	12,29
10,86	11,68	7,71	10,46	11,22	13,56	14,07
6,33	4,96	5,04	5,44	4,60	10,40	10,61
3,49	4,35	4,14	3,79	3,79	12,37	11,99
11,82	10,93	13,30	11,41	10,29	15,15	14,37
6,80	6,37	8,45	5,93	6,40	13,14	14,07
5,43	7,44	6,31	5,76	7,43	10,58	11,14

LF_PCA_3	RF_PCA_1	RF_PCA_2	RF_PCA_3	LH_PCA_1	LH_PCA_2	LH_PCA_3
13,67	13,24	13,92	13,77	15,42	15,13	15,91
11,41	11,00	10,76	12,16	9,68	10,49	11,42
11,94	12,37	11,50	11,30	10,44	10,76	10,81
11,30	11,30	12,83	11,30	8,53	9,16	8,53
13,48	13,45	13,27	13,49	12,31	12,73	12,91
9,62	11,31	12,11	10,76	10,76	10,89	9,68
14,54	14,35	15,68	14,37	15,83	16,60	15,60
12,21	11,30	12,91	12,80	9,82	10,49	11,41
12,31	12,46	12,68	12,57	11,75	12,33	12,56
12,96	13,11	13,52	12,80	16,14	15,68	15,20
11,59	10,42	11,30	11,43	10,86	11,20	11,30
11,94	13,60	10,65	11,07	11,48	11,30	11,30
15,78	15,54	15,74	15,24	13,95	14,76	14,35
14,26	13,32	13,81	13,45	12,91	13,14	12,37
12,51	11,39	11,67	12,47	10,06	11,12	11,66

RH_PCA_1	RH_PCA_2	RH_PCA_3	LF_SPD_1	LF_SPD_2	LF_SPD_3	RF_SPD_1
14,93	15,22	16,37	0,67	0,46	0,43	0,65
9,85	10,92	10,72	0,44	0,43	0,41	0,43
11,30	10,61	11,30	0,48	0,50	0,46	0,48
9,33	9,77	9,33	0,38	0,35	0,38	0,40
12,51	12,038	13,49	0,45	0,62	0,54	0,45
10,44	9,91	9,68	0,43	0,49	0,49	0,41
15,21	17,11	15,20	0,54	0,51	0,55	0,57
10,26	10,49	11,51	0,50	0,48	0,50	0,49
12,27	12,17	13,22	0,34	0,41	0,37	0,35
14,76	15,09	14,73	0,46	0,53	0,63	0,47
10,22	11,60	11,74	0,48	0,56	0,53	0,47
11,30	10,65	9,36	0,41	0,33	0,37	0,39
14,12	14,32	15,06	0,70	0,69	0,56	0,74
12,51	11,70	13,56	0,53	0,42	0,43	0,52
10,22	10,54	12,51	0,50	0,45	0,53	0,47

RF_SPD_2	RF_SPD_3	LH_SPD_1	LH_SPD_2	LH_SPD_3	RH_SPD_1	RH_SPD_2
0,48	0,46	0,61	0,44	0,42	0,61	0,45
0,42	0,40	0,41	0,39	0,38	0,42	0,40
0,54	0,47	0,47	0,49	0,44	0,43	0,53
0,33	0,40	0,40	0,34	0,40	0,37	0,30
0,67	0,54	0,41	0,60	0,51	0,42	0,58
0,48	0,48	0,38	0,45	0,46	0,39	0,45
0,46	0,55	0,51	0,48	0,51	0,54	0,51
0,50	0,49	0,47	0,45	0,47	0,46	0,46
0,41	0,37	0,35	0,43	0,36	0,35	0,41
0,57	0,60	0,46	0,51	0,57	0,43	0,51
0,52	0,50	0,44	0,52	0,47	0,47	0,49
0,35	0,35	0,38	0,29	0,34	0,36	0,33
0,68	0,56	0,67	0,62	0,53	0,66	0,60
0,42	0,42	0,50	0,40	0,39	0,50	0,38
0,43	0,51	0,46	0,43	0,51	0,49	0,43

RH_SPD_3
0,42
0,37
0,45
0,37
0,53
0,43
0,53
0,46
0,36
0,58
0,44
0,33
0,51
0,38
0,50
